# Variation benchmark datasets: update, criteria, quality and applications

**DOI:** 10.1093/database/baz117

**Published:** 2020-02-04

**Authors:** Anasua Sarkar, Yang Yang, Mauno Vihinen

**Affiliations:** 1 Department of Experimental Medical Science, BMC B13, Lund University, SE-22 184 Lund, Sweden; 2 School of Computer Science and Technology, Soochow University, No1. Shizi Street, Suzhou, 215006 Jiangsu, China; 3 Provincial Key Laboratory for Computer Information Processing Technology, No1. Shizi Street, Soochow University, Suzhou, 215006 Jiangsu, China

## Abstract

Development of new computational methods and testing their performance has to be carried out using experimental data. Only in comparison to existing knowledge can method performance be assessed. For that purpose, benchmark datasets with known and verified outcome are needed. High-quality benchmark datasets are valuable and may be difficult, laborious and time consuming to generate. VariBench and VariSNP are the two existing databases for sharing variation benchmark datasets used mainly for variation interpretation. They have been used for training and benchmarking predictors for various types of variations and their effects. VariBench was updated with 419 new datasets from 109 papers containing altogether 329 014 152 variants; however, there is plenty of redundancy between the datasets. VariBench is freely available at http://structure.bmc.lu.se/VariBench/. The contents of the datasets vary depending on information in the original source. The available datasets have been categorized into 20 groups and subgroups. There are datasets for insertions and deletions, substitutions in coding and non-coding region, structure mapped, synonymous and benign variants. Effect-specific datasets include DNA regulatory elements, RNA splicing, and protein property for aggregation, binding free energy, disorder and stability. Then there are several datasets for molecule-specific and disease-specific applications, as well as one dataset for variation phenotype effects. Variants are often described at three molecular levels (DNA, RNA and protein) and sometimes also at the protein structural level including relevant cross references and variant descriptions. The updated VariBench facilitates development and testing of new methods and comparison of obtained performances to previously published methods. We compared the performance of the pathogenicity/tolerance predictor PON-P2 to several benchmark studies, and show that such comparisons are feasible and useful, however, there may be limitations due to lack of provided details and shared data.

Database URL: http://structure.bmc.lu.se/VariBench

## Introduction

Development and testing of computational methods are dependent on experimental data. Only in comparison to existing knowledge can method performance be assessed. For that purpose, benchmark datasets with known and verified outcome are needed. During the last few years, such datasets have been collected for a number of applications in the field of variation interpretation. VariBench (1) and VariSNP (2) are the two existing databases for variation benchmark datasets for variation interpretation. VariBench contains all kinds of datasets while VariSNP is a dedicated resource for variation sets from dbSNP database for short variations (3).

**Figure 1:**
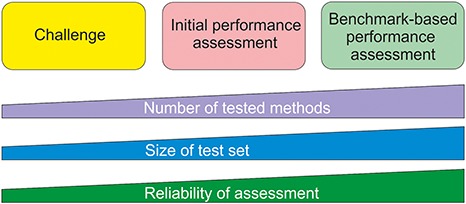
Types of method performance tests. The boxes indicate the three major test settings and the graphs below show how the amounts of certain properties vary along test setup. The figure is adapted from (71).

Benchmark datasets are used both for method training and testing. We can divide testing approaches into three categories (Figure 1). The most reliable are systematic benchmark studies. Quite often the initial method performance assessment is done on somewhat limited test data or does not report all necessary measures. The third group includes studies for initial method and hypothesis testing typically with a limited amount of data. An example for this kind of testing is Critical Assessment of Genome Interpretation (CAGI, https://genomeinterpretation.org/), which has organized several challenges for method developers. These contests with blind data, when the participants do not know the true answer, have been important e.g. for testing new ideas and methods, as well for tackling novel application areas.

High-quality benchmark datasets are valuable and may be difficult, laborious and time consuming to generate. Already from the point of view of reasonable use of resources it is important to share such datasets. Secondly, comparison of method performance is reliable only when using the same test dataset. According to the FAIR principles (4), research data should be made findable, accessible, interoperable and reusable. VariBench and VariSNP provide variation data according to these principles and include relevant metadata.

It is still quite common that authors collect and use extensive datasets for their published papers, but do not share and make the datasets available. This practice prevents others from comparing additional tools to those used in the paper. Even when the data are made available, it may be in a format that makes reuse practically impossible. An example is the datasets used for testing the MutationTaster2 tolerance predictor (5). They were published as figures and at very low resolution. Now, these datasets are available in VariBench.

## Criteria for benchmarks

We defined criteria for a benchmark when the VariBench database was first published (1). These criteria were more extensive than previously used and have been found very useful and still form the basis for inclusion of data and for their representation in VariBench. The criteria are as follows.

*Relevance.* The dataset has to capture the characteristics of the investigated property. Not all available data may be relevant for the phenomenon or may be only indirectly related to it. The collected cases have to be for the specific effect or mechanism under study.

*Representativeness.* The datasets should cover the event space as well as possible, thus preferably containing examples from all the regions relevant to the effect. The actual number of cases for achieving this coverage may vary widely depending on the effect. The dataset should be of sufficient size to allow statistical studies but may not need to include all known instances.

*Non-redundancy*. This means excluding overlapping cases within each dataset.

*Experimentally verified cases.* Method performance comparisons have to be based on experimental data, not on predictions, otherwise the comparison will be about the congruence of methods, not about their true performance.

*Positive and negative cases.* Comprehensive assessment has to be based both on positive (showing the investigated feature) and negative (not having effect) cases.

*Scalability.* It should be possible to test systems of different sizes.

*Reusability.* As datasets are expensive to generate, they should be shared in such a way that they can be used for other investigations. This may mean similar applications or usage in new areas.

Most of the criteria are rather easy to fulfill, but some others are more difficult to take into account. We recently investigated the representativeness of 24 tolerance datasets from VariBench in the human protein universe by analyzing the distribution and coverage of cases in chromosomes, protein structures, CATH domains and classes, Pfam families, Enzyme Commission (EC) categories and Gene Ontology annotations (6). The outcome was that none of the datasets were well representative. When correlating the training data representativeness to the performance of predictors based on them, no clear correlation was found. However, it is apparent that representative training data would allow training of methods that have good performance for cases distributed throughout the event space.

Benchmark studies in relation to variation predictions have been made for variants affecting protein stability (7, 8), protein substitution tolerance/pathogenicity (9–14), protein localization (15), protein disorder (16), protein solubility (17), benign variants (18), transmembrane proteins (19), alternative splicing (20, 21) and phenotypes of amino acid substitutions (22). Many of the datasets used in these studies are available for verification and reuse, but unfortunately e.g. the last one, which is unique, is not accessible.

To test the relevance of the tolerance datasets, we investigated how many disease-causing variations could be found from neutral training data. A small number of such variants were found, 1.13–1.77% (6). These numbers are so small that they do not have a major impact on method performances. VariBench datasets are reusable and scalable, contain experimental cases and are typically non-redundant. However, how redundancy should be defined may depend on the application. For example, when using domain features in variant predictors, variants even in related domain members would be redundant.

## Dataset quality

The quality of benchmark datasets is of utmost significance. This is naturally dependent on the quality of the data sources. There are not many quality schemes in this field. For locus-specific variation databases (LSDBs) there is a quality scheme that contains close to 50 criteria in four main areas including database quality, technical quality, accessibility and timeliness (23). However, these guidelines are not yet widely followed and similar criteria are missing for other types of variation data resources.

Systematics within datasets and databases can significantly improve their quality and usability. For variation data there are a number of systematics solutions available. These include systematic gene names available for human from the HUGO Gene Nomenclature Committee (HGNC) (24), Human Genome Variation Society (HGVS) variation nomenclature (25), Locus Reference Genomic (LRG) and (26) RefSeq reference sequences (27), and Variation Ontology (VariO) variation type, effect and mechanism annotations (28).

Quality relates to numerous aspects in the datasets, the correctness of variation and gene/protein and disease information, relevance of references, etc. We recently selected cases from ProTherm (29) to build an unbiased dataset for the protein variant stability predictor PON-tstab (30). We were aware that the database had some problems, however, were surprised with the extent of problematic cases. While making the selection, we noticed numerous issues, such as cases of two-stage denaturation pathways where values for all the steps and then the total value were provided; there were errors in sequences, variants, recorded measuring temperatures, ΔΔ*G* values and their signs and units, and in indicated PDB structures; and so on. The uncorrected and wrong data have been used for development of tens of prediction methods. This is probably an extreme exception (ProTherm was taken away from the internet after our paper was published); however, this indicates that one has to be careful even when using popular data. When including datasets to VariBench we performed several quality controls, however, we also list datasets that may contain problems e.g. numerous ProTherm sub-selections that have been published and sometimes used in several papers. They have been included for comparative purposes.

## How to test predictor performance

The use of a benchmark dataset is just one of the requirements for systematic method performance assessment. Proper measures are needed to find out the qualities of performance. Most of the currently available prediction methods are binary, distributing cases into two categories. There are guidelines for how to test and report method performance (31–33). There is also a checklist what to report when using such methods in publications.

Results for binary methods are presented in a contingency (also called for confusion) table out of which different measures can be calculated. The most important ones are the following six, which according to the guidelines (32) have to be provided for comprehensive assessments. Specificity, sensitivity, positive and negative predictive values (PPV and NPV) use half of the data in the matrix, while accuracy and Matthews correlation coefficient (MCC) use data from all the four data cells. Additional useful measures include area under curve when presenting Receiver Operating Characteristic curves and Overall Performance Measure. Good methods display a balanced performance and their values for measures differ only slightly.

In case there is an imbalance in the number of cases in the classes, it has to be mitigated (31). Several approaches are available for that. Cases used for testing method performance should not have been used for training them, otherwise there is circularity that overinflates performance measures (14). A scheme has been presented on how datasets should be split for training and testing as well as for blind testing (34). When there are more than two predicted classes additional measures are available (31, 32). In addition to these measures, method assessment can contain other factors such as time required for predictions, as well as user friendliness and clarity of the service and results.

Datasets used for assessment have to be of sufficient size. There are a number of reasons for this requirement. Widely used machine learning methods are statistical by nature and require a relatively large number of cases for reliable testing. If we think the event space, in the case of human proteins, there are 380 different amino acid substitution types, 150 of which are more likely due to emerging because of a single nucleotide substitution within the coding region for a codon. These substitutions can appear in numerous different contexts, thus too small test datasets should be avoided. There are several performance assessments, especially for variants in a single protein or a small number of genes/proteins that do not have any statistical power. The smallest dataset we have seen contained just nine substitutions, based on which a detailed analysis was performed to recommend the best performing tools!

Variation interpretation is often carried out in relation to human diseases. It is important to note that diseases are not binary states (benign/disease) instead there is a continuum and certain disease state can appear due to numerous different combinations of disease components, see the pathogenicity model (35). This aspect has not been taken into account in benchmark datasets apart from the training data for PON-PS (36) and clinical data for cystic fibrosis (37).

## Variation datasets

We have collected from literature, websites and databases datasets, which have been used for training and benchmarking various types of variations and their effects (Table 1). The new datasets come from 109 papers. There are 419 new separate datasets containing altogether 329 014 152 variants. One paper can contain more than one dataset. The number of unique variants is smaller as many of the datasets are different subsets of commonly used datasets such as ClinVar or ProTherm or VariBench itself. The total number is dominated by VariSNP cases. The original VariBench version contained 17 datasets from 10 articles representing five variation categories, thus the growth in the database size has been substantial.

**Table 1:** New benchmark datasets added to VariBench

**Origin of data**	**Dataset first used for**	**Number of variants in each dataset**	**Number of different genes, transcripts or proteins in each dataset**	**Reference**
**Variation type datasets**				
Insertions and deletions (0/0)				
HGMD, 1000 GP	DDIG-In	659, 2008, 2479, 3861, 579, 2008, 2413, 3861	659, 737, 2447, 751, 1122, 493, 1996, 1933, 2989	(74)
ClinVar, 1000 GP, ESP6500 SIFT-Indel	ENTPRISE-X	6513,5023,82, 366, 3171, 1604, 181, 1025	1078, 1361, 38, 307, 2491, 1251, 170, 1018	(75)
SwissProt, 100 GP, SM2PH	KD4i	2734	1973	(76)
Sequence alignments	SIFT Indel	474, 9710	474, 9698	(77)
Substitutions, coding region (6/10)				
*Training datasets*				
Literature, patents	PredictSNP	10 581, 5871, 43 882, 32 776, 3497, 11 994	12, 12, 11 410, 8336, 1421, 23	(11)
HGMD, SwissProt	FATHMM, FATHMM-XF	69 141, 94 995, 69 141	12 412, 47 510, 12 412	(78, 79)
ClinVar, HGMD	MutationTaster	2600, 2199, 1100, 1100	617, 1652, 618, 1006	(5)
HumDiv, UniProt, ClinVar	VIPUR	9477, 1542, 382, 949, 4992, 6555	2444, 1477, 381, 913, 4362, 1120	(80)
Humsavar	BadMut	33 483	8185	(81)
HumVar, ExoVar, VariBenchSelected, SwissVarSelected	RAPSODY	21 946	2728	(73)
ClinVar, ESP	DANN	16 627 775, 49 407 057	–, –	(48)
SwissProt	NetSAP	5375, 1152	218, 734	(82)
VariBench	PON-P2	10 717, 13 063, 1108, 1605, 6144, 8661, 656, 1053	980, 5936, 93, 669, 786, 4522, 75, 518	(10)
Humsavar, VariBench	SuSPect	18 633, 64 163	6874, 12 171	(83)
CMG, DDD, ClinVar, ExoVar, 1000 GP, Hg19, Gencode, ESP6500	MAPPIN	64, 158, 3595, 15 702, 512 370, 51 599, 11 763, 1 048 544	27, 100, 961, 309, −, 3888, 10 035, −	(84)
Uniprot, 1000 GP, literature, VariBench, ARIC study	Ensemble predictor	36 192, 238, 19 520, 7953, 33 511, 26 962	35 892, 237, 19 427, 7907, 33 305, 26 829	(85)
ClinVar	PhD-SNP^g^	48 534, 1408	43 273, 1407	(86)
Multiple gene panel	MVP	1161	10	(69)
ADME genes				
LoF only	ADME optimized	337, 180	43, 43	(68)
CinVar, NHGRI GWAS catalog, COSMIC, VariSNP	PredictSNP2	25 480, 12 050, 142 722, 16 716, 71 674	9929, 5570, −, 5949, 19 702	(87)
*Test datasets*				
HumVar, ExoVar, VariBench, predictSNP, SwissVar	Circularity	40 389, 8850, 10 266, 16 098, 12 729	9250, 3612, 4203, 4456, 5057	(14)
ClinVar, literature, PredictSNP	ACMG/AMP rules	14 819, 1442, 4667, 6931, 5379, 12 496, 14 819, 4192, 16 064, 10 308, 7766	1726, 75, 476, 1695, 1146, 1723, 1821, 656, 15 921, 4183, 1349	(51)
ClinVar, TP53, PPARG	Performance assessment	11 995	3717	(49)
UniProt	Guideline discordant/PRDIS	28 474, 336 730	2393, 2388	(52)
ESP6500, HGMD	Compensated pathogenic deviations	1964	685	(53)
VariBench	Representativeness	446 013, 23 671, 19 335, 19 459, 14 610, 17 623, 17 525, 14 647, 13 096, 13 069, 12 584, 1605, 1301, 8664, 7152, 1053, 751, 16 098, 10 266, 8850, 40 389, 21 151, 22 196, 75 042	53 850, 8762, 1190, 7816, 1100, 6047, 954, 5476, 884, 4998, 980, 546, 93, 3800, 786, 425, 75, 4456, 4201, 3612, 9250, 8791, 1852, 12 735	(6)
*Structure mapped variants*				
PDB, UniProt	PON-SC	349, 7795	62, 4574	(54)
3D	3D structure analysis	374	334	(55)
LSDBs, literature, ClinVar	Membrane proteins	2058	2019	(19)
*Synonymous*				
ClinVar, GRASP, GWAS Catalog, GWASdb, PolymiRTS, PubMed, Web of Knowledge	dbDSM	2021	954	(88)
dbDSM, ClinVar, literature	IDSV	600, 5331	493, 99	(89)
*Benign*				
dbSNP	VariSNP	446 013, 956 958, 470 473, 3802, 9285, 3402, 5277, 11 339, 588, 318 967, 1 804 501, 610 396, 25 930 776	19 597, 51 764, 19 618, 2972, 7242, 1056, 1542, 8444, 584, 48 018, 35 200, 39 531, 65 437	(2)
ExAX	Assessment of benign variants	63 197, 1302	37 148, 400	(18)
**Effect-specific datasets**				
DNA regulatory elements				
Ensembl Compara, 1000 GP	Pathogenic regulatory variants	42, 142, 153, 43, 65, 3, 5	19, 58, 72, 24, 3, 1, 3	(90)
OMIM, ClinVar, VarDi, GWAS Catalog, HGMD, COSMIC, FANTOM5, ENCODE	Regulatory variants	27 558, 20 963, 43 364	3826, 6653, 40 548	(91)
dbSNP, HGMP, HapMap, GWAS Catalog	Regulatory elements	225, 241 910	66, 19 346	(92)
ENCODE, NIH Roadmap Epigenomics	CAPE	7948, 4044, 2693, 51, 156, 56 497, 2029	4744, 3214, 1980, 48, 112, 43 676, 1568	(93)
Whole-genome sequences, GiaB, HGMD, ClinVar	CDTS	15 741, 427, 10 979, 67 144 812, 34 687 974, 30 634 572, 31 893 124, 61 372 584	1862, 309, −, −, −, −, −	(94)
Literature, OMIM, Epi4K	TraP	402, 97, 103	64, 97, 102	(95)
HGMD, 1000GP, ClinVar	ShapeGTP	4462, 1116	1362, 691	(96)
ClinVar, literature	NCBoost	655, 6550, 770	612, 6380, 765	(97)
RNA splicing (1/1)				
Literature, LSDBs, HGP	DBASS3 and DBASS5	307, 577	131, 166	(40, 41)
HGMD, SpliceDisease database, DBASS, 1000 GP	dbscSNV	2959, 45, 2025	2938, 2, 333	(21)
Experimental	BRCA1 and BRCA2	13, 15, 33, 38, 35, 73	1, 1, 1, 1, 1, 1	(98)
Ensembl, UCSC Genome Browser	HumanSplicingFinder	424, 81, 15, 89	222, 6, 4, 8	(99)
HGMD	MutPred Splice	2354, 638	452, 176	(100)
hg19, GenBank, dbSNP	ASSEDA	41, 8, 12	14, 7, 11	(101)
Experimental	*RB1*	3, 17, 13, 6	1, 1, 1, 1	(102)
Experimental	*LDLR*	18, 18	1, 1	(103)
Experimental	*BRCA1* and *BRCA2*	6, 29, 6, 19	2, 2, 2, 1	(104)
Experimental, LSDBs	*BRCA1* and *BRCA2*	53, 4, 4, 6, 5	2, 2, 2, 2, 2	(105)
Experimental	*BRCA1* and *BRCA2*	24, 22, 13, 10, 10, 5, 11	2, 2, 2, 2, 2, 5, 2	(106)
Experimental	Exon 1^st^ nucleotide	25, 5, 9, 5, 5, 9, 30, 9	20, 5, 9, 20, 4, 7, 24, 7	(107)
ClinVar, 1000GP	Splice site consensus region	222, 50	138, 44	(108)
Protein aggregation (0/0)				
WALTZ-DB, AmylHex, AmylFrag, AGGRESCAN, TANGO	AmyLoad	1400	–	(38)
Experimental	WALTZ-DB	1089	140	(39)
Binding free energy				
Literature, ASEdb, PIN, ABbind, PROXiMATE, dbMPIKT	SKEMPI 2.0	7085	348	(42)
SKEMPI	Flex ddG	1249	55	(109)
Protein disorder (0/0)				
Literature	PON-Diso	103	32	(16)
Protein solubility (0/0)				
Literature	PON-Sol	443	61	(17)
Protein stability (4/6)				
*Single variants*				
ProTherm	PON-Tstab	1564	80	(30)
ProTherm	I-Mutant2.0	2087, 1948	58, 64	(110)
ProTherm	Average assignment	1791, 1396, 2204	70, 45, 89	(111)
ProTherm	iPTREE-STAB	1859	64	(112)
ProTherm	SVM-WIN31 and SVM-3D12	1681, 1634, 499	58, 55, 34	(113)
ProTherm	PoPMuSiC-2.0	2648	132	(114)
ProTherm	sMMGB	1109	60	(115)
ProTherm	M8 and M47	2760, 1810	75, 71	(116)
ProTherm	EASE-MM	238, 1676, 543	25, 70, 55	(117)
ProTherm	HoTMuSiC	1626	101	(118)
	SAAFEC	1262, 983	49, 28	(119)
ProTherm	STRUM	3421, 306	148, 32	(120)
ProTherm	Metapredictor	605	58	(121)
ProTherm	Automute	1962, 1925, 1749	77, 54, 64	(122)
TP53	TP53	42	1	(123)
ProTherm	S^sym^	684	15	(124)
ProTherm, experimental data, ASEdb	Alanine scanning for binding energy	768, 2971, 1005, 380, 2154	56, 119, 82, 19, 84	(125)
ProTherm	Rosetta	1210	75	(126)
*Double variants*				
ProTherm	WET-STAB	180	28	(127)
**Molecule-specific datasets** (1/2)				
InSiGHT	PON-MMR2	178, 45	5, 5	(61)
Literature	PON-mt-tRNA	145	22	(56)
BTKbase	PON-BTK	152	1	(60)
Kin-Driver, ClinVar, Ensembl	Kinact	384, 258	42, 23	(57)
Literature	KinMutBase	1414	39	(43)
COSMIC	Kin-Driver	783, 648	43, 43	(44)
OMIM, KinMutBase, HGMD	Protein kinases	1463, 999, 302	392, 49, 144	(59, 128)
UniProt, KinMutBase, SAAPdb, COSMIC	wKin-Mut	865, 2627	447, 65	(58)
dbSNP, HGMD, COSMIC, literature	PTENpred	676	1	(129)
UniProt, Humsavar	Protein-specific predictors	1 872 222 in 82 files	82	(12)
Literature	SAVER	187	1	(130)
Literature, experimental, dbSNP, ExAC, ESP	DPYD-Varifier	69, 295	1, 1	(131)
Experimental	*BRCA1/2*	201, 68	2, 2	(132)
Experimental	CFTR	20, 11	1, 1	(37)
CHAMP, literature	HApredictor	1138	1	(133)
Humsavar	MutaCYP	29, 285, 328	4, 15, 36	(134)
UniProt, HGMD, MutDB, dbSNP, literature	KvSNP	1259, 176	87, 60	(135)
**Disease-specific datasets** (0/0)				
Literature, TP53 database, ClinVar, DoCM	Pan-cancer analysis	659, 65, 387	33, 60, 1	(64)
Literature, IARC TP53 Database, UMD BRCA1 and BRCA2	Cancer	3706	15	(65)
ICGC, TCGA, Pediatric Cancer Genome Project, dbSNP	Cancer	4690	17	(66)
Literature, LOVD, Inherited Arrhythmia Database	Long QT syndrome	90, 82, 8, 81, 113, 99, 14, 58, 55, 52, 28, 24, 109, 101, 8, 312	1, 1, 1, 3, 1, 5, 1, 1, 1, 3, 2, 3, 1, 1, 1, 7	(62)
Experimental	PolyPhen-HCM	74, 78 983	6, 6	(63)
Functional assays	FASMIC	1049, 95, 40, 785, 21, 14, 35, 65, 22	93, 95, 38, 57, 6, 8, 14, 22, 9	(136)
Literature	dbCPM	941	161	(45)
cBioPortal, COSMIC, MSK-IMPACT cohort	OncoKB	4472	595	(47)
TCGA	DoCM	1364	132	(46)
**Phenotype dataset** (0/0)				
Literature, LSDBs	PON-PS	2527, 401	83, 8	(36)

VariBench datasets are freely available at http://structure.bmc.lu.se/VariBench/ and can be downloaded separately. The website contains basic information about the datasets, their origin and for what purpose they were initially used for. There is also information about in how many genes, transcripts or proteins the variants appear. Datasets are categorized similar to Table 1 for easy access. The contents of the datasets vary depending on information in the original source. We have enriched many of them e.g. by mapping to reference sequences or PDB structures and some contain VariO annotations. Columns in the original sources irrelevant for VariBench were removed.

**Figure 2:**
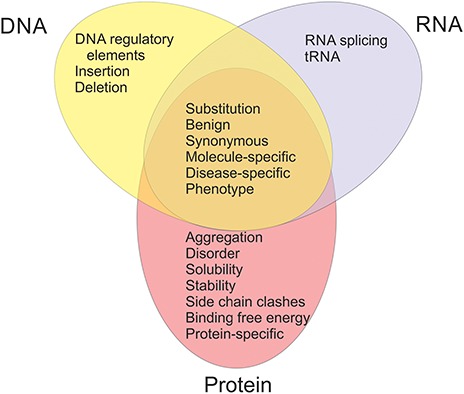
Types of benchmark datasets and their relations in VariBench.

The available datasets have been categorized into 20 groups and subgroups as indicated in Figure 2. The figure shows also the relationships of the datasets in different categories. Variants are often described at three molecular levels (DNA, RNA and protein) and sometimes also at protein structural level, including relevant cross references and variant descriptions. VariBench utilizes and follows a number of standards and systematics including HGVS variation nomenclature, HGNC gene names (not in all databases due to mapping problems) and VariO annotations in some datasets.

Links are available to data in some external databases, including AmyLoad (38) and WALTZ-DB (39) for protein aggregation, DBASS3 and DBASS5 (40, 41) for splicing variants, SKEMPI (42), cancer datasets in KinMutBase (43), Kin-Driver (44), dbCPM (45), DoCM (46), OncoKB (47) and tolerance predictor training set in DANN (48). The latter has a link due to its huge size, the others since they are databases and as such easy to use directly and updated by third parties. We excluded datasets used in CAGI experiments, since they are available for registered participants only. LSDBs were excluded because data from these sources usually have to be manually selected before using as benchmark. Most of the time, they do not contain clear information for variant relevance to disease(s). Datasets for structural genomic variants were excluded, because they usually lack information about exact variation positions.

Unfortunately, many papers, even those reporting on benchmarking, do not contain and share the data, which does not allow others to extend the analyses and reuse the datasets.

### Variation type datasets

Variation types include insertions and deletions, coding and non-coding region substitutions, which are divided into training and test datasets, structure mapped variants, as well as synonymous, and benign variants. There are now data from four amino acid insertion effect predictors, mainly for short alterations. Only datasets added after the release of the first version of VariBench are discussed here. In Table 1 is shown how many datasets and publications in each category appeared in the first edition.

Training datasets have mainly been used for development of machine learning predictors, there are 17 new datasets. They typically also contain test sets. Six test datasets have been specifically designed for method performance assessments. These include a set for addressing circularity (14) and pathogenicity/tolerance method performance assessment (49). The American College of Medical Genetics and Genomics (ACMG) and the Association for Molecular Pathology (AMP) has published guidelines for variant interpretation (50). These include instructions for use of prediction methods. A dataset was obtained for addressing concordance of prediction methods (51). Another study addressed discordant cases (52). Protein sequences of even closely related organisms contain differences and some of these are compensated variants where a disease-related variant in human is normal in another organism due to additional alteration(s) at other site(s). A dataset has been collected for such variants (53). Unfortunately, only the benign variants were made available. Analysis of the dataset representativeness, how well the datasets represent the variation space, was investigated for 24 datasets in VariBench and VariSNP (6). These cases were mapped to reference sequence and are now available in the database.

Variations are mapped into protein 3D structures in several datasets. Dedicated datasets contain those used for developing a method for predicting side-chain clashes because of residue substitutions (54), analysis of effects on structures and functions of substitutions (55) and investigation of variations in membrane proteins (19).

There are two datasets for synonymous variants as well as two for benign ones.

### Effect-specific datasets

These datasets are for various types of effects. On DNA level there are eight sets for DNA regulatory elements, and on RNA level 14 datasets for splicing. Most of the splicing datasets are very small, but there are a few with substantially larger numbers. In the first version of VariBench, there were only protein stability datasets in this category, totally six datasets from four studies.

Many more sets are available for effects on protein level. Protein aggregation (two datasets), binding free energy (2), disorder (1), solubility (1) and stability are the currently available categories. Among protein stability datasets, there are 22 new datasets for single variants, almost all originating from ProTherm, and one dataset for double variants.

### Molecule-specific datasets

There are in VariBench 18 specific datasets for certain molecules. There is a set of variants used to train PON-mt-tRNA for substitutions affecting mitochondrial transfer RNA (tRNA) molecules (56). This is of special interest as there are 22 unique mitochondrial tRNAs that are implicated in a number of diseases.

The other datasets are protein specific. Kinact (57), Kin-Driver (44), KinMutBase (43), Kin-Mut (58) and the protein kinase dataset (59) contain variation information for protein kinases. The PON-BTK dataset was used to train a predictor for protein kinase domain variants in Bruton tyrosine kinase (BTK) (60). There is a set for mismatch repair (MMR) proteins MLH1, MSH2, MSH6 and PMS2 and used to train PON-MMR2 (61).

Single amino acid substitutions were collected in 82 proteins to test whether there is a difference in performance for protein specific and generic predictors (12). All the datasets contain at least ~100 variants. The results indicated vast differences in performances, the best generic predictors outperforming the specific predictors in most but not all cases.

The remaining datasets in this category are for variants in individual genes/proteins.

### Disease-specific datasets

This category contains totally nine datasets, six of which are for cancer, one for long QT syndrome (62) and another for hypertrophic cardiomyopathy (63).

Although there are numerous studies of cancer variations, the functional verification of the relevance of those variants for the disease is usually missing. VariBench contains three datasets for variants in cancer, which have been experimentally tested (64–66), and links to three other sources, namely dbCPM (45), DoCM (46) and OncoKB (47). In addition, there is the FASMIC dataset for variants that are largely cancer related (67).

### Phenotype dataset

One dataset contains information for disease phenotype, whether there is mild/moderate or severe disease due to substitutions. This dataset was used to train disease severity predictor called PON-PS (36).

## Benchmark use case

VariBench datasets have mainly been used for prediction method development and testing. As the benchmark studies typically have not contained all the best performing tools, we compared the performance of the variant tolerance/pathogenicity predictor PON-P2, since this tool has been the best or among the best performing methods in a number of previous investigations (10, 12, 18, 19, 52). The setup was similar in all these studies to test the outcome of a spectrum of methods. We extended the published benchmark studies by repeating the original analyses with PON-P2. To avoid circularity, we first excluded from the datasets all cases that had been used for training PON-P2. The results are shown in Table 2 and are reported according to the published guidelines (32) and including some additional measures.

**Table 2:** Performance of PON-P2 on test datasets

**Dataset**	**TP**	**FP**	**TN**	**FN**	**Coverage**	**PPV**	**NPV**	**Sens**	**Spec**	**Acc** ^a^	**MCC**	**OPM**
MutationTaster2, ClinVar (5)	544	9	959	32	0.685	0.99	0.947	0.944	0.991	0.968	0.936	0.910
MutationTaster2 (5)	407	10	803	63	0.635	0.986	0.881	0.866	0.988	0.927	0.860	0.810
Circularity, PredictSNPSelected (14)	5116	341	3173	590	0.623	0.940	0.770	0.900	0.860	0.880	0.730	0.606
Circularity, SwissVarSelected (14)	1551	818	3194	773	0.557	0.650	0.810	0.670	0.800	0.750	0.460	0.325
ACMG/AMP, MetaSVM (51)	2588	364	2457	192	0.503	0.878	0.927	0.931	0.871	0.901	0.803	0.733
ACMG/AMP, ClinVar_balanced (51)	841	136	608	69	0.455	0.835	0.915	0.924	0.817	0.871	0.746	0.666
ACMG/AMP, VaribenchSelected_Tolerance (51)	1727	171	2996	57	0.513	0.947	0.967	0.968	0.946	0.957	0.914	0.875
ACMG/AMP, predictSNPdsel (51)	3752	317	3071	427	0.539	0.906	0.899	0.898	0.906	0.902	0.804	0.734
ACMG/AMP, ClinVar_Sep2016 (51)	1050	215	1726	102	0.514	0.892	0.909	0.911	0.889	0.900	0.801	0.729
ACMG/AMP, Dominant_Recessive_Genes (51)	1284	98	619	52	0.506	0.875	0.957	0.961	0.863	0.912	0.828	0.769
ACMG/AMP, Oncogenes_TSG (51)	535	59	74	3	0.497	0.692	0.99	0.994	0.556	0.908 0.775(AN)	0.613	0.559
Variants in 3D structures (73)	5077	300	1060	266	0.337	0.812	0.94	0.95	0.779	0.865	0.741	0.676
ClinVar dataset (49)	1040	157	1200	169	0.541	0.881	0.864	0.86	0.884	0.872	0.745	0.664
TP53 dataset (49)	430	130	13	3	0.509	0.522	0.929	0.993	0.091	0.769 0.542(AN)	0.195	0.269
PPARG dataset (49)	131	1376	7	0	0.598	0.501	1.000	1.000	0.005	0.503	0.000	0.111
Cancer, functionally tested (65)	561	18	16	3	0.605	0.653	0.989	0.995	0.471	0.965 0.733(AN)	0.546	0.523
Cancer, non-COSMIC functionally tested (65)	108	10	14	3	0.455	0.700	0.956	0.973	0.583	0.904 0.778(AN)	0.604	0.549

The exercise indicated that reproducibility and reusability could not be achieved in a number of cases due to problems in reporting. We had to exclude some published benchmark studies. The dataset for pharmacogenetics variants (68) was too small for reliable estimation. The paper for compensated variants (53) did not share the disease-related variants, and thus could not be evaluated. Of the dataset used by Qian *et al*. (69) only 36 cases were not included to the PON-P2 training set, and therefore the benchmark had to be excluded because of too small size.

We were able to perform the analysis for six studies and we analyzed altogether 17 datasets. Full comparison was not possible in all cases as some details were not available. Therefore, we discuss and compare the performances based on the information in the original papers, but list all the details from our study in Table 2.

For MutationTaster2 the published test data has not been previously available due to being in a format that prevents reuse of the data. MutationTaster 2 was originally compared to five tools and versions (MutationTaster1, PolyPhen humdiv and humvar, PROVEAN and SIFT) (5). The accuracy and specificity are better for PON-P2 than the scores for the six tested tools and sensitivity is the second best. Only the measures given in the original article are discussed in here.

The study of circularity problems in variant testing was conducted on predictSNPSelected and SwissVarSelected datasets (14). The performance of PON-P2 is superior compared to the eight tested predictors (MutationTaster2, PolyPhen, MutationAssessor, CADD, SIFT, LRT, FatHMM-U, FatHMM-W, Gerp++ and phyloP). In the test for predictSNPSelected dataset, NPV, PPV, sensitivity, accuracy and MCC are the best for PON-P2. Only for specificity, it is the second best predictor with a margin of 1%. In the data for SwissVarSelected, PON-P2 has the best score for PPV, accuracy and MCC. It is the second best for NPV and specificity, by 1–2% margin to the best, and for sensitivity. On both datasets, PON-P2 showed the most balanced performances.

Twenty-five tools were tested according to ACMG/AMP guidelines using several datasets (51). The compared methods were REVEL, VEST3, MetaSVM, MetaLR, hEAt, Condel, MutPred, Mcap, Eigen, CADD, PolyPhen2, PROVEAN, SIFT, EA, MutationAssessor, MutationTaster, phyloP100way, FATHMM, DANN, LRT, SiPhy, phastConst100way, GenoCanyon, GERP and Integrated_fitCons. Unfortunately, the results were not comprehensively reported. The paper contains data for AUC scores but they are presented as figures. The exact values were difficult to estimate, especially when results for 18 datasets were combined into single figures. In the end, we performed the test for eight of these datasets. In the ClinVar balanced data the AUC of PON-P2 is either shared first or second, and in VariBenchselected data it has the best performance. Comparison for the six other datasets is not as reliable, but we can summarize that the PON-P2 performance is among the best if not the best for all of these. It is unfortunate that exact numbers were not provided by the authors.

The performances of 23 methods (FATHMM, fitCons, LRT, MutationAssessor, MutationTaster, PlyPhen humdiv and humvar versions, PROVEAN, SIFT, VEST3, GERP++, phastCons, phyloP, SiPhy, CADD, DANN, Eigen, FATHMM-MKL, GenoCanyon, M-CAP, MetaLR, MetaSVM and REVEL) were tested on three datasets: ClinVar and two protein-specific sets for TP53 and PPARG (49). They had also a fourth set for autism spectrum diseases, but since there is no experimental evidence for the relation of these variations to the disease, that set was excluded. Although the study was well performed and described, it seems that the authors have not corrected for class imbalance. For the methods to be comparable the measures should be calculated based on the same data and have equal numbers of positive and negative cases. If that is not the case, the imbalance has to be mitigated with one of the available solutions. Some of the other benchmark studies may suffer from the same problem, but we are not sure due to incomplete descriptions of the studies. None of the tools can predict all possible variations and thus they have predictions for different numbers. Therefore we present the results both for non-normalized and normalized data. We believe that the former was used by the authors. In the case of ClinVar data, PON-P2 has better PPV, accuracy and MCC than the other methods tested in the paper.

In the case of TP53 data, the PON-P2 accuracy is second best when the data are not normalized; on other measures, PON-P2 is ranked the fourth or worse. All cancer variants, such as those in TP53, were excluded from the PON-P2 training data. This was done because the effects of variations in cancers usually have not been experimentally verified. A variant in TP53 is not ‘pathogenic’ alone, several variants in different proteins are needed for cancer.

All the predictors are known to have variable performance depending on the tested protein, see the study of protein-specific predictors (12); this study showed that PON-P2 had better performance for 85% of proteins, being the best of the five tested tools (PolyPhen-2, SIFT, PON-P2, MutationTaster2, CADD). PPARG seems to be another example for which PON-P2 has poor performance (49). An additional reason for poor performance may be that the PPARG data is not for pathogenicity, instead it is a ‘function score’ that is based on the distribution of FACS sorted cells (70). The same applies to the TP53 test data which is based on the protein function, not pathogenicity. Depending on a protein, the threshold for phenotype can be anything between 1% and 85% of the wild type activity (Vihinen, in preparation). We have previously tested PON-P2 in protein function prediction but with poor (71) or mixed (72) outcome. This is because the method has not been trained and intended for this task. These results indicate the importance of applying computational tools to their intended purpose or at least testing the performance carefully before applied to new tasks.

Another study tested the performance of 14 tools (SEQ + DYN, SEQ, DYN, MutationTaster2, PolyPhen2, MutationAssessor, CADD, SIFT, LRT, FATHMM-U, Gerp++, phyloP, Condel and Logit) in relation to structural dynamics, which was used as a proxy for functional significance of amino acid substitutions (73). PON-P2 has the best sensitivity, specificity, NPV and MMC, it is the second best for accuracy but only 13th for PPV. The explanation for the latter observation is that many of the tested tools are severely biased, having very high PPV but very low NPV, whereas the performance of PON-P2 was again balanced over all the measures.

The exercise indicated that it is possible to compare predictors to published results based on exactly the same datasets. The new performance results for PON-P2 are in line with several previously published studies that have indicated the method to be a top performer on different benchmarks (10, 12, 18, 19, 52). When choosing a method(s), one should look at consistent performance over several benchmarks.

Full comparisons were not always possible because of incomplete performance assessments. Therefore, authors should meticulously describe all details and procedures in the data analysis as well as share the datasets used. Even if the data is taken from public sources, it is not possible for others to obtain exactly the same dataset as used in the papers even when applying the same selection criteria, as some important aspects seem always to be missing. In summary, it was possible to compare performances for methods not included into original studies. This is important in many ways and contributes toward increased reproducibility and comparability. Good datasets are difficult to obtain, therefore VariBench will serve as a hub for sharing these important data.
